# Cerebral Pathology and Cognition in Diabetes: The Merits of Multiparametric Neuroimaging

**DOI:** 10.3389/fnins.2017.00188

**Published:** 2017-04-05

**Authors:** Frank C. G. van Bussel, Walter H. Backes, Paul A. M. Hofman, Robert J. van Oostenbrugge, Martin P. J. van Boxtel, Frans R. J. Verhey, Harry W. M. Steinbusch, Miranda T. Schram, Coen D. A. Stehouwer, Joachim E. Wildberger, Jacobus F. A. Jansen

**Affiliations:** ^1^Department of Radiology, Maastricht University Medical CenterMaastricht, Netherlands; ^2^School for Mental Health and Neuroscience, Maastricht University Medical CenterMaastricht, Netherlands; ^3^Department of Neurology, Maastricht University Medical CenterMaastricht, Netherlands; ^4^Cardiovascular Research Institute Maastricht, Maastricht University Medical CenterMaastricht, Netherlands; ^5^Department of Psychiatry and Neuropsychology, Maastricht University Medical CenterMaastricht, Netherlands; ^6^Department of Internal Medicine, Maastricht University Medical CenterMaastricht, Netherlands

**Keywords:** type 2 diabetes mellitus, magnetic resonance imaging, cognition, functional MRI, multiparametric MRI

## Abstract

Type 2 diabetes mellitus is associated with accelerated cognitive decline and various cerebral abnormalities visible on MRI. The exact pathophysiological mechanisms underlying cognitive decline in diabetes still remain to be elucidated. In addition to conventional images, MRI offers a versatile set of novel contrasts, including blood perfusion, neuronal function, white matter microstructure, and metabolic function. These more-advanced multiparametric MRI contrasts and the pertaining parameters are able to reveal abnormalities in type 2 diabetes, which may be related to cognitive decline. To further elucidate the nature of the link between diabetes, cognitive decline, and brain abnormalities, and changes over time thereof, biomarkers are needed which can be provided by advanced MRI techniques. This review summarizes to what extent MRI, especially advanced multiparametric techniques, can elucidate the underlying neuronal substrate that reflects the cognitive decline in type 2 diabetes.

## Introduction

Type 2 diabetes mellitus is a common metabolic disorder, characterized by chronic hyperglycemia, in a context of insulin resistance and relative insulin deficiency (Gispen and Biessels, 2000). Type 2 diabetes has commonly been considered a disease of elderly populations. However, with today's unhealthy lifestyle, also an increasing number of younger (that is, middle-age) people are developing diabetes.

Type 2 diabetes has a broad range of serious clinical complications, including nephropathy, retinopathy, and cardiovascular disease, and is often accompanied by cardiovascular risk factors such as hypertension and dyslipidemia. Hyperglycemia damages a selection of cell types, including neurons, which are unable to reduce the transport of glucose inside the cell, leading to high glucose (Brownlee, 2005). Type 2 diabetes is also associated with cognitive deficits, accelerated cognitive decline, an increased risk of dementia, and Alzheimer disease (AD) (Biessels et al., 2006). In type 2 diabetes, cognitive changes mainly affect learning, memory and information processing speed (Cheng et al., 2012). For recent reviews on cognition and type 2 diabetes, the reader is referred to specific recent reviews by Koekkoek et al. (2014) and Geijselaers et al. (2015).

In recent years, numerous studies have highlighted the adverse effects of diabetes on brain physiology and cognitive function to assess contributing pathophysiological mechanisms (Biessels and Reijmer, 2014; Brundel et al., 2014a). Most studies have applied conventional MRI with multiple contrasts to detect macrostructural cerebral changes. However, macrostructural abnormalities on MRI reflect end-stage effects of impaired tissue, and conventional MRI is probably not sensitive enough to detect the earliest cerebral changes, expectedly more closely reflecting mechanisms, associated with cognitive decline (Tofts, 2003). For this purpose, potentially more-sensitive MRI techniques, such as functional MRI (fMRI) and diffusion MRI (dMRI), can be used, which could lead to a better insight into the mechanisms that precede macrostructural (end-stage) abnormalities.

**Figure 1:**
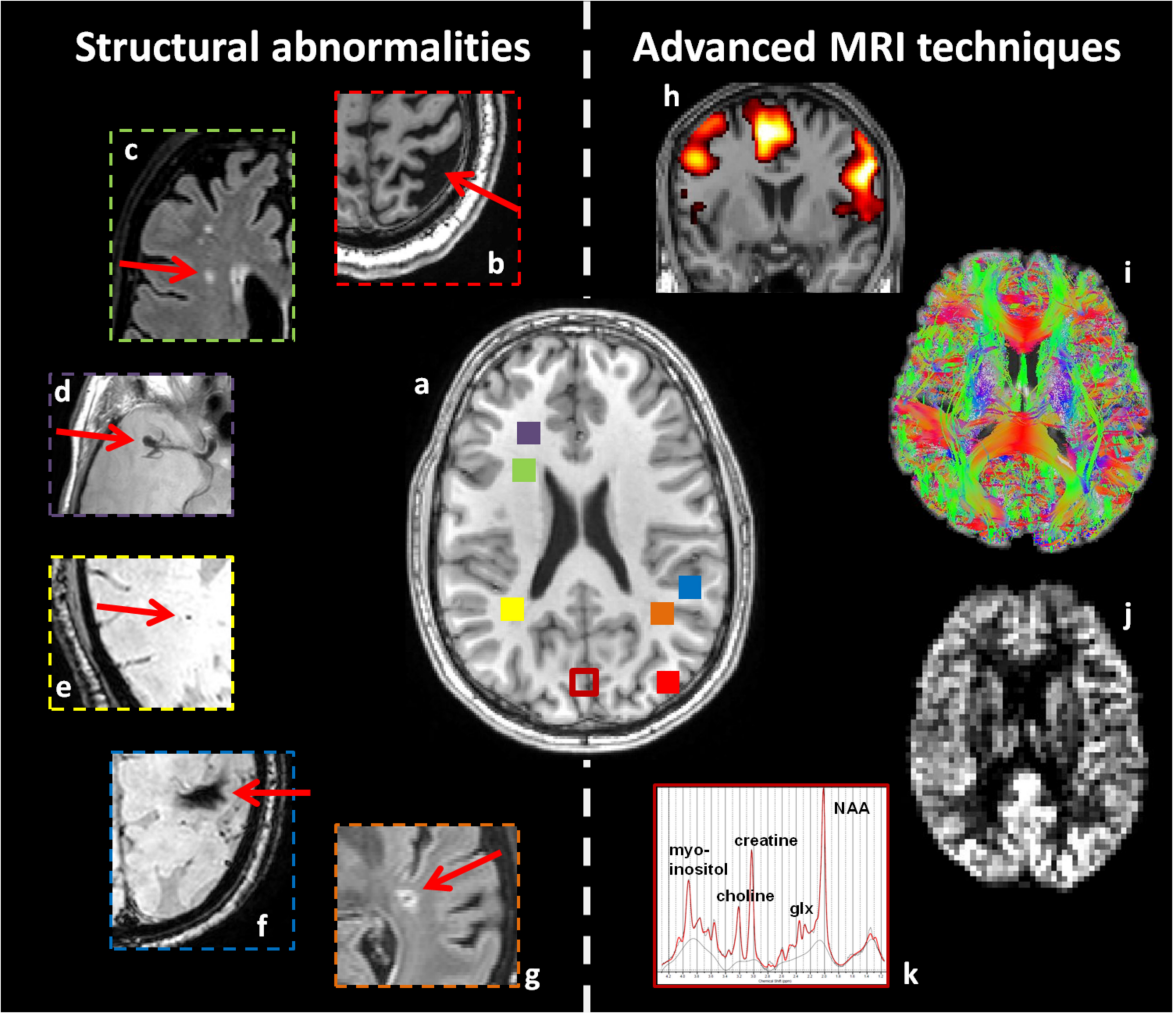
**Overview of structural abnormalities which may be found in patients with type 2 diabetes (b–g)**, and advanced MRI techniques sensitive to more subtle cerebral alterations **(h–k)**. This figure is an illustration from the authors' clinic. **(a)** T1-weighted image of a healthy young brain. Structural abnormalities in patients with type 2 diabetes, as highlighted with red arrows. **(b)** Atrophy (T1WI), **(c)** white matter lesions (FLAIR), **(d)** aneurysm (T2WI), **(e)** microbleeding (T2^*^-weighted), **(f)** macrobleeding (T2^*^-weighted), and **(g)** lacunar infarct (FLAIR). Advanced MRI techniques: **(h)** fMRI, **(i)** dMRI, **(j)** arterial spin labeling, and **(k)** MRS. Corresponding colored squares in **(a)** represent the approximate location where the structural abnormalities were found and where the single voxel for spectroscopy was located, respectively.

The present narrative review summarizes recent literature and provides an overview of the various brain abnormalities associated with type 2 diabetes in combination with cognitive decrements. The aim is to provide the available evidence for neuronal substrates of cognitive impairment in type 2 diabetes. It will explore the appropriate MRI techniques to study associations with cognitive performance in patients with type 2 diabetes (for an overview of typical abnormalities and the corresponding techniques, see Figure 1), and will make recommendations for future research. This review is structured according to the various types of cerebral abnormalities and the appropriate MRI techniques available to study pathophysiology, in the range from routine clinical application to explorative research.

## Atrophy

Cerebral atrophy can generally be defined as the shrinkage of brain tissue, which is a result of neurodegenerative processes, such as the loss of neurons and their interconnections (Jobst et al., 1994). Many studies on type 2 diabetes, using various structural MRI techniques, report on atrophy (den Heijer et al., 2003; de Bresser et al., 2010; van Elderen et al., 2010). Associations have been found between brain atrophy and decreased performance in various cognitive domains (Tiehuis et al., 2009; Hayashi et al., 2011; Moran et al., 2013; Zhang Y. et al., 2014), including memory, attention and executive function, as well as processing speed, motor speed, and sensory speed. Also the progression of atrophy was found related to cognitive decrements in type 2 diabetes (van Elderen et al., 2010; Reijmer et al., 2011).

## Small vessel disease

Cerebral small vessel disease (cSVD) can be generally defined as pathological processes with various etiologies that affect the small arteries, arterioles, venules, and capillaries of the brain (Wardlaw et al., 2013). Signs of cSVD are white matter lesions, microbleeds, silent brain infarcts and lacunar abnormalities, which are also indicative for cognitive decline (Imamine et al., 2011).

### White matter lesions

White matter lesions (WMLs) are typically observed as regions of bright, high-signal intensity in the white matter (i.e., white matter hyperintensities) depicted on T2-weighted and, especially, FLAIR images (Wardlaw et al., 2013). The underlying pathophysiology of WMLs is still poorly understood and is assumed to include multiple factors of vascular (through ischemia or arteriosclerosis) or inflammatory (through transudation of CSF) origin (Fazekas et al., 1998).

WMLs are often divided in periventricular WMLs, which are located close to the ventricles, and deep WMLs, which are located in subcortical gray matter (Wardlaw et al., 2013). It was shown that periventricular, but not subcortical, WMLs are associated with the rate of cognitive decline in elderly non-demented individuals (De Groot et al., 2002).

Numerous studies report on WMLs in patients with type 2 diabetes (Manschot et al., 2006; Jongen et al., 2007; van Harten et al., 2007; Imamine et al., 2011). More specific, deep (subcortical) WMLs, periventricular WMLs, and WMLs in general are found in patients with type 2 diabetes. WMLs are also related with impaired cognition in type 2 diabetes (Manschot et al., 2006; Jongen et al., 2007; van Harten et al., 2007; Imamine et al., 2011), especially in the domains of processing speed, memory, attention and executive functioning, and motor speed.

### Microbleeds

Cerebral microbleeds result from focal leakages of small blood vessels (Wardlaw et al., 2013). They are thought to contain iron deposits (Wardlaw et al., 2013). Typically, microbleeds are found only incidentally on MRI, but are thought to play an important role in cognitive decline (Wardlaw et al., 2013). The reported prevalence of microbleeds increases with age (Imamine et al., 2011). Microbleeds do not seem to be associated with type 2 diabetic patients with cognitive impairment (Moran et al., 2013), which is also confirmed at high field (7T) (Brundel et al., 2014b).

### Silent brain infarcts

Silent brain infarcts (SBIs) are clinically asymptomatic (i.e., they lack stroke-like symptoms), but visible (generally 2–5 mm in diameter) as focal lesions on MRI, and are associated with cognitive deficits that commonly remain unnoticed (vermeer et al., 2007).

Patients with type 2 diabetes often display SBIs, which are also related to impaired cognitive performance (Manschot et al., 2006; Imamine et al., 2011). The number of SBIs and/or progression of SBIs are especially linked to decrements in motor speed, attention and executive functioning (Imamine et al., 2011; Umegaki et al., 2011).

### Lacunar abnormalities

Lacunes are pathologically defined as small areas (3–15 mm in diameter) of infarction, which is a result from an occlusion of one of the small penetrating branches of large cerebral arteries (Wardlaw et al., 2013) and are associated with cognitive impairment (Schneider et al., 2003). In type 2 diabetes, lacunar infarcts often progress (van Harten et al., 2006; Umegaki, 2010), likely caused by ischemia (Imamine et al., 2011).

Cerebral infarcts (i.e., lacunar, cortical, subcortical infarcts, or infarcts in general) have been observed in patients with type 2 diabetes (Manschot et al., 2006; Moran et al., 2013). Most studies report a relationship between cerebral infarcts and decreased performance in various cognitive domains, including processing speed, sensory speed, memory, executive function, and global cognition.

For the detection of cerebral atrophy or cSVD, various structural MRI techniques have been used. However, these techniques cannot unravel more subtle details of tissue alterations that underlie or precede the atrophy or cSVD. For this, more-advanced MRI techniques can be used, which will be discussed below.

## Impaired cerebral perfusion

Cerebral perfusion is defined as the amount of blood flowing through a definite volume of tissue in a given time (Filippi et al., 2010) and can be estimated using Arterial Spin Labeling (ASL) and Intravoxel Incoherent Motion (IVIM) imaging, or measured globally using a velocity-sensitive, phase-contrast MRI technique (Tiehuis et al., 2008; Brundel et al., 2012b; Novak et al., 2014; Rusinek et al., 2015; van Bussel et al., 2015; Jansen et al., 2016). The ASL technique is based on magnetic labeling of arterial blood (e.g., blood in the common carotid artery), which is used as an temporary endogenous tracer in the brain. The IVIM technique enables assessment of both the parenchyma and microvasculature and is based on the diffusion of water molecules in parenchyma and incoherent motion of water molecules in the microvasculature. The velocity-sensitive, phase-contrast MRI technique is based on differences in phase of the magnetic spins. An advantage of using ASL or IVIM is that these techniques make it possible to investigate regional differences related to disease pathology instead of only a gross measurement of total brain perfusion with phase-contrast MRI (Le Bihan et al., 1986; Ryan et al., 2014).

A fair comparison between ASL and IVIM is not trivial due to the different complex physical mechanisms that contribute to the detected signal. However, the former is a truly quantitative method, which has been validated with PET (positron emission tomography) perfusion measurements (van Golen et al., 2014), whereas the latter is yet more experimental, though it can provide a higher signal-to-noise ratio (SNR), and the possibility for an increased spatial resolution.

Thus far, some studies, one using phase contrast MRI (1.5T) (Tiehuis et al., 2008) and one using ASL (3T) (Rusinek et al., 2015), did not find any differences in global perfusion between patients with type 2 diabetes and controls, while other studies (ASL, Novak et al., 2014; Xia et al., 2015a and IVIM, van Bussel et al., 2015, all at 3T) observed regional differences in perfusion. Possibly, differences in MRI methodology could explain these conflicting results. Atrophy can be a big confounder when assessing hypoperfusion using ASL, indeed most results disappear after correction for atrophy (Jansen et al., 2016). A recent ASL study applied a new analysis approach tallying the “distributed deviating voxels,” and hypoperfusion was found in patients with type 2 diabetes, which remained significant after correction for atrophy in the subcortical gray matter (Jansen et al., 2016).

One phase contrast MRI study observed a positive association between perfusion and cognition, but this study was not able to explain the link of diabetes with cognitive performance (Tiehuis et al., 2008). Some studies did find a relationship between perfusion and impaired cognition in patients with type 2 diabetes (Brundel et al., 2012b; Xia et al., 2015a), although another study did not find this relationship (Jansen et al., 2016). Promising results regarding reduced cerebral perfusion in the insula cortex and cognitive performance were shown in a pilot ASL study (Novak et al., 2014). After insulin administration, memory and verbal fluency improved, and perfusion was elevated in the insula cortex of participants with diabetes, suggesting the involvement of an insulin mechanism. In type 2 diabetes, perfusion of the global gray matter was positively associated with verbal fluency (Rusinek et al., 2015), although local hippocampal perfusion (as measured using IVIM) had a negative association with memory performance (van Bussel et al., 2015). These results suggest the involvement of a vascular mechanism, and that the association might be dependent on the brain region.

Taken together, all perfusion techniques observed a relation with cognitive performance, which highlights the link between a vascular mechanism and cognitive decline. However, to observe regional differences in perfusion, the more-advanced MRI techniques (i.e., ASL and IVIM) appear more sensitive to contribute to the understanding of cognitive decline in patients with type 2 diabetes.

## Neuronal dysfunction

Neuronal dysfunction refers to all impairments of the neuronal system, including reduced functional activity of certain brain regions and connectivity between different regions (Zhou et al., 2010). Functional MRI (fMRI) offers the opportunity to investigate to which extent neuronal regions are active, in terms of blood oxygenation changes. The underlying principle is that neuronal activity leads to locally increased blood flow and oxygenation. Previous studies using the amplitude of low frequency fluctuations (ALFF), a measure of spontaneous neuronal activity, regional homogeneity, a measure of the neural regional synchronization, and functional connectivity, assessed by correlating time signals from distinct brain regions, reported on abnormal brain activity in patients with type 2 diabetes (Zhou et al., 2010; Musen et al., 2012; Xia et al., 2013; Cui et al., 2014).

### Functional connectivity

Reduced functional connectivity in the default mode network (DMN), i.e., the network of active brain regions when the brain is at rest and the participant is not focusing on anything particular, has been observed in patients with type 2 diabetes (Zhou et al., 2010; Musen et al., 2012; Chen et al., 2014, 2015, 2016; Hoogenboom et al., 2014; Cui et al., 2015; Xia et al., 2015b; Zhang H. et al., 2015). Moreover, reduced functional connectivity between the hippocampus and widespread regions in the DMN (Zhou et al., 2010), including the medial frontal cortex (Zhang H. et al., 2015) has been reported, in addition to reduced functional connectivity between the posterior cingulate and the medial frontal gyri and other regions in the DMN (Musen et al., 2012; Hoogenboom et al., 2014). Furthermore, reduced connectivity within the attention networks has been described (Xia et al., 2015b), which was associated with neuropsychological scores and glycated hemoglobin. Reduced connectivity of the DMN was related to impaired memory (Zhou et al., 2010; Zhang H. et al., 2015), executive function (Zhou et al., 2010), verbal fluency (Zhang H. et al., 2015), and lower global cognition (Zhang H. et al., 2015). The disrupted functional connectivity in the DMN has been shown to be inversely correlated with insulin resistance (Musen et al., 2012) in type 2 diabetes, hinting at an underlying insulin-related mechanism. This thought is enhanced by the observation of acutely increased functional connectivity between the hippocampus and multiple regions in the DMN after intranasal insulin administration (Zhang H. et al., 2015).

Interestingly, it was recently shown that participants with type 2 diabetes displayed altered fMRI network measures, characterized by a higher efficiency, compared with control participants (van Bussel et al., 2016c). Also subjects with pre-diabetes were studied, whose network measures fell between those with diabetes and control participants. The authors suggested that functional reorganization of the cerebral networks might act as a compensatory mechanism for cognitive decrements (van Bussel et al., 2016c).

### Signal fluctuations

ALFF and regional homogeneity alterations have been reported in a variety of DMN brain regions (including temporal lobe and frontal lobes) in patients with type 2 diabetes (Xia et al., 2013; Cui et al., 2014; Zhou et al., 2014). The altered ALFF and regional homogeneity values were related to impaired cognition, especially in the domains of attention and executive function (Xia et al., 2013; Cui et al., 2014; Zhou et al., 2014), speed (Xia et al., 2013; Cui et al., 2014), memory (Cui et al., 2014), and global cognition (Zhou et al., 2014). Moreover, ALFF values in the middle temporal gyrus were also inversely related to glycated hemoglobin (Xia et al., 2013) and insulin resistance in the diabetic group was negatively correlated with altered neuronal activity (Cui et al., 2014).

### Brain activation

Altered brain activation has also been found in patients with type 2 diabetes during a memory task, especially in task-related regions of the DMN (Marder et al., 2014), frontal cortex (Chen et al., 2014; Marder et al., 2014; He et al., 2015), parietal cortex (He et al., 2015) and the fronto-parietal network (Zhang Y. et al., 2016). Moreover, functional activation or connectivity is not only associated with memory performance (Zhang Y. et al., 2016), but also insulin resistance (Marder et al., 2014; Xia et al., 2015c), glycated hemoglobin (Marder et al., 2014; He et al., 2015), plasma glucose (Marder et al., 2014), and cholesterol (Xia et al., 2015d), suggesting a major role of glucose and lipid metabolism.

Overall, all functional MRI studies consistently show evidence of altered neuronal activity or functional connectivity in patients with type 2 diabetes and cognitive decrements.

## White matter tract abnormalities

White matter tract abnormalities refer to impaired integrity or altered organization of axonal bundles and can be investigated using diffusion MRI (dMRI). This technique is based on diffusion of water molecules, and during the dMRI acquisition, tissue is sensitized with the local characteristics of molecular diffusion. The measures most often analyzed by dMRI are fractional anisotropy (FA) and apparent diffusion coefficient (ADC). FA is a measure of tract directionality and ADC is a measure of water diffusivity. Clinically, an increase in ADC has been associated with reduced (neuronal) cell packing and increased extracellular space, possibly due to failure of neurogenesis or cell loss (Eriksson et al., 2001). Recently, analysis methods have become available that allow the assessment of the integrity and efficiency of structural networks, using graph theoretical analysis on dMRI data (Reijmer et al., 2013b).

### Local alterations

Microstructural abnormalities have been published for various brain regions in type 2 diabetes (Yau et al., 2009, 2010; Falvey et al., 2013; Zhang J. et al., 2014, 2016; van Bussel et al., 2016b; Xiong et al., 2016). Reduced FA has been observed in the white matter (Yau et al., 2010; Falvey et al., 2013) mostly concentrated in frontal and temporal regions (Yau et al., 2009), while elevated ADC values were found in a number of brain regions, including the hippocampus (Falvey et al., 2013) and multiple gray matter regions (Yau et al., 2010). Temporal lobe abnormalities were associated with impaired memory (Yau et al., 2009; van Bussel et al., 2015).

### Network alterations

Altered network and structural connectivity in type 2 diabetes have been shown using tractography (Reijmer et al., 2013a,b; Hoogenboom et al., 2014; van Bussel et al., 2016b; Yang et al., 2016; Zhang J. et al., 2016). Local and global network properties (i.e., cluster coefficient, global efficiency, path length) were altered and associated with impaired processing speed (Reijmer et al., 2013b). Elevated ADC and reduced FA were found in different tracts, including the superior longitudinal fasciculus (Reijmer et al., 2013a), uncinate fasciculus (Reijmer et al., 2013a; Hoogenboom et al., 2014), inferior longitudinal fasciculus (Reijmer et al., 2013a), corpus callosum (Reijmer et al., 2013a), and cingulum bundle (Hoogenboom et al., 2014). These tract abnormalities were associated with impaired processing speed and memory (Reijmer et al., 2013a; Hoogenboom et al., 2014) and highlight an underlying glucose-mediated mechanism as glycated hemoglobin and fasting blood glucose were also related to these tract abnormalities (Hoogenboom et al., 2014). Moreover, altered hippocampal white matter connectivity appear to be associated with memory decrements and type 2 diabetes (van Bussel et al., 2016b).

Diffusion MRI studies implicate that patients with type 2 diabetes show evidence of white matter microstructure, tract, and network abnormalities.

## Metabolic dysfunction

Proton magnetic resonance spectroscopy (^1^H-MRS) enables the assessment of metabolic changes through the identification and quantification of spectral peaks associated with tissue metabolites (Jansen et al., 2006). ^1^H-MRS is often used to investigate N-acetylaspartate (NAA), Choline (Cho), Creatine (Cr), myo-inositol (mIns), γ-aminobutyric acid (GABA), and glutamate. NAA is a measure of neuronal integrity and a surrogate marker of normal functioning neurons. Cho is an indirect marker of myelination and cell membrane metabolism. Cr is a measure of energy metabolism, and mIns has been proposed as a glial marker and as an end-product of persistent hyperglycaemia (Jansen et al., 2006). GABA and glutamate are major inhibitory and exhibitory neurotransmitters, respectively. However, *in vivo* detection and quantification of these neurotransmitter concentrations at low field strengths (<3T) are complicated due to spectral overlap with other metabolites. Another relevant metabolite in the context of diabetes is glucose, which typically requires high field strengths (>3T) for reliably detection with ^1^H-MRS (Gruetter et al., 1996). An alternative method to study brain glucose levels using MR spectroscopy is ^13^C-MR spectroscopy (van De Ven et al., 2012).

MR spectroscopy studies on type 2 diabetes in relationship with cognition have thus far been proven to be challenging, and often no associations between metabolic alterations and cognitive performance were found (Haroon et al., 2009; Tiehuis et al., 2010). However, a recent study found higher GABA+ levels in participants with type 2 diabetes, and higher GABA+ levels in participants with both high HbA1c levels and less cognitive performance (van Bussel et al., 2016a). The authors concluded that participants with type 2 diabetes have alterations in the GABAergic neurotransmitter system, which are related to lower cognitive functioning, which hints at the involvement of an underlying metabolic mechanism.

## Interpretation

Table 1 provides an overview of all studies on type 2 diabetes, in which cognitive performance is related to diverse cerebral MRI contrasts. From this it can be appreciated that neuroradiologically visible MRI biomarkers (atrophy, WMLs, and lacunar abnormalities) and more subtle abnormalities (impaired cerebral perfusion, neuronal dysfunction, and white matter tract abnormalities) are related to cognitive decline, with a striking agreement between studies. For the other abnormalities (including microbleeds, SBIs, and metabolic dysfunction) the evidence of relationships with cognition is less convincing.

**Table 1:** **Overview of neuroimaging abnormalities associated with cognitive performance in type 2 diabetes mellitus**.

**Brain abnormalities**	**MRI techniques**	**Major outcomes**	**References**
**CLINICAL APPLICATIONS**			
Atrophy	– T1WI – T2WI – FLAIR – IR	Cerebral atrophy increases with cognitive decline	den Heijer et al., 2003; Manschot et al., 2006; van Elderen et al., 2010; Hayashi et al., 2011; Reijmer et al., 2011
**SMALL VESSEL DISEASE**			
White matter lesions	– T2WI – FLAIR	White matter lesion load increases with cognitive decline	Manschot et al., 2006; Jongen et al., 2007; van Harten et al., 2007; Imamine et al., 2011
Microbleeds	– T2^*^WI	No evidence of microbleeds with cognitive decline	Moran et al., 2013; Brundel et al., 2014b
Silent brain infarcts	– T1WI – T2WI – FLAIR	Progression of silent brain infarcts seems related to cognitive decline	Imamine et al., 2011; Umegaki et al., 2011
Lacunar abnormalities	– T1WI – T2WI – FLAIR	Cerebral ischemic lesions are related to cognitive decline	Manschot et al., 2006; Umegaki, 2010
Impaired cerebral perfusion	– ASL – PC-MRI – IVIM	Diverse results regarding perfusion in diabetes. Perfusion related to cognitive decline	Tiehuis et al., 2008; Brundel et al., 2012b; Novak et al., 2014; Rusinek et al., 2015; Xia et al., 2015a; van Bussel et al., 2015; Jansen et al., 2016
**NEURONAL DYSFUNCTION**			
Functional connectivity	– fMRI (connectivity)	Reduced functional connectivity in relationship with cognition; higher efficiency in T2DM with cognitive decrements	Zhou et al., 2010; Xia et al., 2015b; Zhang Y.-W. et al., 2015; van Bussel et al., 2016c
Signal fluctuations	– ALFF	Altered ALFF related to impaired cognition	Xia et al., 2013; Cui et al., 2014; Zhou et al., 2014
Brain activation	– fMRI (activation)	Altered neuronal activity in relationship with cognitive decline	Zhang Y. et al., 2016
**WHITE MATTER TRACT ABNORMALITIES**			
Local alterations	– dMRI (diffusion measures)	Temporal lobe abnormalities were associated with impaired memory	Yau et al., 2009; van Bussel et al., 2016b
Network alterations	– dMRI (connectivity)	Tract abnormalities and network alterations related to impaired cognition	Reijmer et al., 2013a,b; Hoogenboom et al., 2014; van Bussel et al., 2016b
Metabolic dysfunction	– MRS	Insufficient evidence regarding metabolic alterations and cognitive performance	Haroon et al., 2009; Tiehuis et al., 2010; van Bussel et al., 2016a

Only MRI references in combination with cognitive performance are included in this table. T2(^*^)WI, T2(star)-weighted images; IR, inversion recovery images; ASL, arterial spin labeling; PC-MRI, (velocity-sensitive) phase-contrast MRI; IVIM, intravoxel incoherent imaging; fMRI, functional MRI; ALFF, amplitude of low frequency fluctuations; dMRI, diffusion MRI; MRS, magnetic resonance spectroscopy.

Most studies are associated with various methodological limitations. Most notably, often only a limited number of subjects is included. Furthermore, the studies show a pronounced diversity regarding subject selection, matching of subjects, diagnosis and classification of diabetes, adjustment for risk factors, and data analysis methods. Due to the different designs and limited number of available studies, it is difficult for studies reporting negative results to assess whether the applied techniques (or study methods) are not sensitive enough to pick up cognitive performance-related alterations, or whether these alterations are not present at all. Interestingly, in those studies where cerebral changes were found, these were most often located in the frontal and/or temporal lobe (den Heijer et al., 2003; Zhou et al., 2010, 2014; Musen et al., 2012; He et al., 2015; van Bussel et al., 2015), which is in close agreement with the type of cognitive decline typically experienced in type 2 diabetes (Gold et al., 2007).

Type 2 diabetes is also known to increase the risk of developing AD (Steen et al., 2005; Cheng et al., 2012). MRI studies show that gray matter loss, insulin resistance, and medial temporal lobe atrophy are associated with AD (Thompson et al., 2003; Biessels et al., 2006), traits also present in patients with type 2 diabetes (den Heijer et al., 2003). These results suggest that diabetes might to some extent be linked to Alzheimer's Disease (AD) and that diabetes and AD might share similar mechanisms underlying cognitive decline (Ryan et al., 2014).

## Future outlook

As the neuronal mechanisms underlying cognitive decline associated with type 2 diabetes still remain to be elucidated, and studies using more-advanced and potentially more-sensitive MRI techniques are scarce, intensified research is needed to investigate the underlying mechanisms of brain damage (Jouvent et al., 2010). It will also be interesting to investigate cognitive decline in pre-diabetic stages such as the metabolic syndrome or impaired glucose mechanism (Grundy, 2006; van Bussel et al., 2016c).

In addition to the imaging techniques discussed in this review, other novel MRI approaches might also yield interesting new biomarkers, such as Dynamic Contrast Enhanced MR Imaging, which is an MRI technique where T1-weighted scans are acquired dynamically after injection of a contrast agent, and pharmacokinetic modeling of the enhancing tissue signal can provide information about physiological tissue characteristics, including BBB integrity in terms of leakage of contrast medium (Taheri et al., 2011). It could be relevant to study the role of BBB in diabetes, because disruption of the BBB is also considered to be a result of cSVD.

Furthermore, metabolites that are relatively difficult to detect, such as GABA, dedicated MRS spectral editing sequences exist to identify and quantify these metabolite concentrations (Puts and Edden, 2012). The use of a specifically designed MRS acquisition scheme allows for the selective recording of signals only from the desired metabolite, while other metabolites are eliminated.

Another important direction is the application of high field MRI (Brundel et al., 2012a), as most studies in this review were performed at 1.5T. High field MRI (≥3T) has several benefits as it provides higher spatial resolution and improved SNR, although it is more susceptible for artifacts. Additionally, future studies should incorporate a multiparametric approach, to provide a more complete picture of the locations and nature of affected cerebral regions. Also, analysis approaches for fMRI and dMRI should focus on cerebral networks, as cognitive functions affected by diabetes correspond to networks, rather than localized brain regions.

Additionally, future, preferably large multicenter studies, are required to validate current findings, or provide a more definitive answer regarding issues for which currently contradictory findings have been reported in different studies (such as the inconsistencies reported regarding type 2 diabetes and global perfusion). For this, quantitative measures are essential, regarding both quantitative MRI as neuropsychological tests to characterize and define in more detail the cognitive status of the population under investigation.

## Clinical relevance

The application of neuroimaging techniques to study diabetes associated accelerated cognitive decline is relevant as we expect to obtain new insights regarding affected brain regions, networks, and tissue abnormalities. Furthermore, MRI measures might provide early biomarkers for cognitive decline (see Table 1 for an overview), and could potentially be used to identify patients at risk. Follow-up studies can be performed to confirm that subjects with sufficient cerebral MRI alterations eventually develop cognitive problems, and one could consider an interventional study with a combination of diet, exercise or medication (Zhang H. et al., 2015) to explore whether cerebral MRI alterations also delay, or even improve, after intervention (Raji et al., 2015). Hence, by performing advanced neuroimaging, a more complete picture can be obtained of the effect of diabetes on the brain, it might provide a better timing of (preventive) therapy, and it could shed some light on the course and efficacy of the therapy to prevent or halt cognitive decline.

## Conclusions

Cognitive decline in type 2 diabetes is associated with brain alterations, which can be detected using neuroimaging. The battery of MRI techniques available to study this topic is highly versatile, and several aspects of brain function and integrity can be studied noninvasively. Advanced, novel MRI techniques are expected to reveal more subtle brain alterations compared with only structural MRI. Therefore, more-advanced multiparametric MRI techniques should be implemented in future studies to investigate the role of diabetes on cognitive performance, and the underlying pathophysiological mechanisms.

## Literature search

We searched PubMed for articles published until September 19, 2016, with the following terms and combinations of these terms: “arterial disease,” “arterial spin labeling,” “atrophy,” “axon damage,” “brain,” “cerebral,” “cogniti^*^,” “connectivity,” “diabet^*^,” “diffusion tensor imaging,” “DTI,” “fMRI,” “functional MRI,” “imaging,” “lacun^*^,” “lacunar infarct,” “microbleeds,” “microstructural abnormalit^*^,” “MRI,” “MRS,” “MR spectroscopy,” “neuronal dysfunction,” “neuronal function,” “neuropathy,” “perfusion,” “syndrome,” “type 2,” “vessel disease,” “white matter lesion.”

We included articles identified from these searches and relevant references cited in the articles.

The neuropsychological terminology is subdivided in (1) (verbal) memory, (2a) (information) processing speed, (2b) sensory speed, (2c) motor speed, (3) IQ, (4) global cognition, (5) attention functions, (6) executive functions, (7) psychomotor functions, (8) visuoconstruction, and (9) fluency, according to Hebben and Milberg (2009). Speed is subdivided into three components: (1) processing speed (central part/brain), (2) sensory speed (visual aspects) and (3) motor speed (conducting part of a test/trail).

Animal studies, studies on patients with type 1 diabetes mellitus, and studies in which MRI results were presented without addressing correlations with cognitive performance were not included. Only articles written in English were included.

## Author contributions

FCGvB searched for published reports and wrote the first draft of the Review. WB and JJ helped to improve the first draft with addition of relevant reports, suggestions for structure of the Review, and the idea for a schematic table. PH, Rv, Mv, FRJV, HS, MS, CS, and JW read the Review critically and made suggestions for improvements.

## Funding

JJ was funded by VENI research grant 916.11.059 from The Netherlands Organization for Scientific Research (NWO) and The Netherlands Organization for Health Research and Development (ZonMw). Additionally, this work was supported by “Stichting de Weijerhorst” foundation.

### Conflict of interest statement

The authors declare that the research was conducted in the absence of any commercial or financial relationships that could be construed as a potential conflict of interest.
